# Automatic multilabel detection of ICD10 codes in Dutch cardiology discharge letters using neural networks

**DOI:** 10.1038/s41746-021-00404-9

**Published:** 2021-02-26

**Authors:** Arjan Sammani, Ayoub Bagheri, Peter G. M. van der Heijden, Anneline S. J. M. te Riele, Annette F. Baas, C. A. J. Oosters, Daniel Oberski, Folkert W. Asselbergs

**Affiliations:** 1 Department of Cardiology, Division of Heart & Lungs, University Medical Centre Utrecht, University of Utrecht, Utrecht, The Netherlands; 2 Department of Methodology and Statistics, Faculty of Social Sciences, Utrecht University, Utrecht, The Netherlands; 3 Southampton Statistical Sciences Research Institute, University of Southampton, Highfield, Southampton, UK; 4 Department of Genetics, Division Laboratories, Pharmacy and Biomedical Genetics, University Medical Centre Utrecht, University of Utrecht, Utrecht, The Netherlands; 5 Department of Information and Finance, Division of Health Administration and Information, University Medical Centre Utrecht, Utrecht, The Netherlands; 6 Institute of Cardiovascular Science, Faculty of Population Health Sciences, University College London, London, UK; 7 Health Data Research UK, Institute of Health Informatics, University College London, London, UK

**Keywords:** Health care, Diseases, Health services

## Abstract

Standard reference terminology of diagnoses and risk factors is crucial for billing, epidemiological studies, and inter/intranational comparisons of diseases. The International Classification of Disease (ICD) is a standardized and widely used method, but the manual classification is an enormously time-consuming endeavor. Natural language processing together with machine learning allows automated structuring of diagnoses using ICD-10 codes, but the limited performance of machine learning models, the necessity of gigantic datasets, and poor reliability of terminal parts of these codes restricted clinical usability. We aimed to create a high performing pipeline for automated classification of reliable ICD-10 codes in the free medical text in cardiology. We focussed on frequently used and well-defined three- and four-digit ICD-10 codes that still have enough granularity to be clinically relevant such as atrial fibrillation (I48), acute myocardial infarction (I21), or dilated cardiomyopathy (I42.0). Our pipeline uses a deep neural network known as a Bidirectional Gated Recurrent Unit Neural Network and was trained and tested with 5548 discharge letters and validated in 5089 discharge and procedural letters. As in clinical practice discharge letters may be labeled with more than one code, we assessed the single- and multilabel performance of main diagnoses and cardiovascular risk factors. We investigated using both the entire body of text and only the summary paragraph, supplemented by age and sex. Given the privacy-sensitive information included in discharge letters, we added a de-identification step. The performance was high, with F1 scores of 0.76–0.99 for three-character and 0.87–0.98 for four-character ICD-10 codes, and was best when using complete discharge letters. Adding variables age/sex did not affect results. For model interpretability, word coefficients were provided and qualitative assessment of classification was manually performed. Because of its high performance, this pipeline can be useful to decrease the administrative burden of classifying discharge diagnoses and may serve as a scaffold for reimbursement and research applications.

## Introduction

**Fig. 1:**
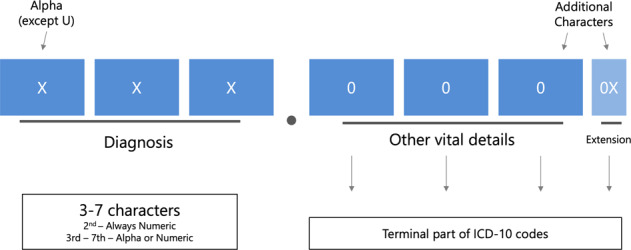
The alphanumeric structure of ICD-10 codes. The codes may be three to seven characters and terminal parts of these codes describe the diagnoses in more detail. The first character (Alpha) is considered to be the “chapter” of ICD-10 coding, followed by a digit.

**Fig. 2:**
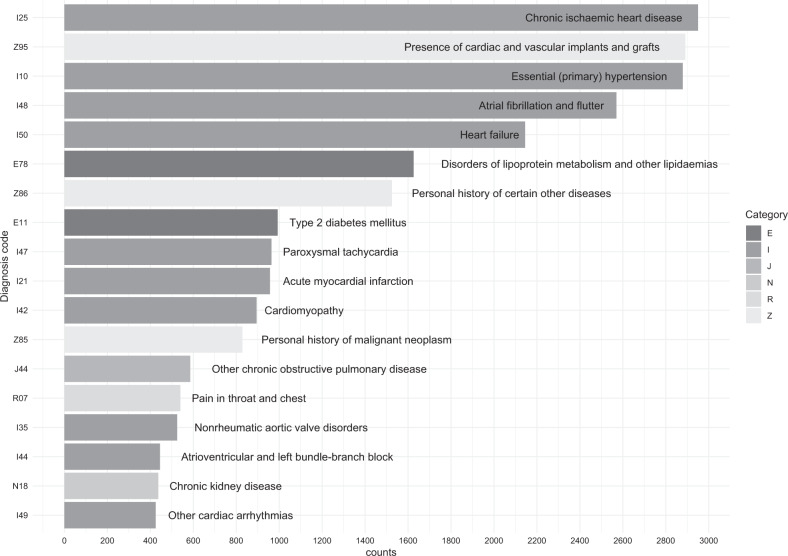
Codes with more than 400 appearances in the dataset. Three character codes are presented on the y-axis and their labels are provided in the figure. These codes are derived from letters from the department of cardiology.

Electronic health records (EHRs) enable fast information retrieval and contain both structured (e.g., laboratory values and numeric measurements) and unstructured data (free text in clinical notes)^1^. Clinical discharge letters are an important source of information, but the translation from free text to structured data remains challenging^2^. To structure diagnoses, the international classification of diseases (ICD-10) coding system was created. This classification system is hierarchical and multiple codes may be assigned to a single discharge letter (multilabel). ICD-10 is alphanumerically structured, with seven possible digits arranged hierarchically as shown in Figs. 1 and 2^3^. The classification is performed by practitioners, managers or medical coders and serves worldwide in clinical practice (e.g., medical history and billing), research (e.g., trial recruitment), and (inter)national epidemiological studies^2–5^. Manual classification is an enormously costly endeavor, its quality depends on the expertise of who is performing the classification task and the reliability for terminal parts of ICD-10 codes can be poor, even among trained medical coders^5^.

Natural language processing (NLP) together with machine learning allows automating ICD-10 coding for discharge letters^2^. This task is particularly challenging because of: (i) the unstructured nature of free text, (ii) the multilabel setting of ICD10 codes, and (iii) the large number of terminal ICD-10 codes^4^. Several attempts have been made to automatically assign ICD-10 codes to medical documents ranging from rule-based to machine learning approaches^2,6^. Generally speaking, rule-based methods have good performance, which is however restricted to conditions that seldomly occur in free-text clinical notes (given possibly ambiguous wording/spelling, multilabel classification and sparsity). Machine-learning techniques on the other hand have shown increasingly promising results^2,4,6,7^. Supervised classification can often be simplified by considering only top-level “chapters” of the ICD-10 hierarchy or by only considering a single label or disease groups as output. By doing so, some models do not depict a real-world situation and are less applicable in daily clinical practice^4,7–16^.

More recently, multilabel classification of detailed ICD-10 codes has been improved greatly with deep learning, showing better performance when using RNNs. These improved models however rely on enormous labeled datasets (Table 1)^2,4,6,17^. Unsupervised or semi-supervised classification algorithms are not dependent on curated EHR datasets and may even reduce bias from practice and coding behavior. Recent work by Sonabend et al. illustrated an unsupervised knowledge integration algorithm by using pre-existing clinical knowledge sources such as Medscape and mapped identified terms to concept unique identifiers. This resulted in a well-performing classification algorithm for six entities^8^. In general, clinically relevant granularity in predicted labels and reliability of terminal parts of ICD-10 codes is challenging to model performance (Table 1)^2,4,6–16^. Contextual word embeddings (ELMo and BERT) are derived from pretrained bidirectional language models and show substantial performance improvements in many NLP tasks^18,19^. Fine-tuning of these pretrained models is, given the language and context of the training data, in essence, efficient and performant but poses challenges when contextual embeddings in a subdomain and language are lacking^6,18,20^. Furthermore, patient privacy may be compromised if these language models are published online^21^.

**Table 1:** Performance of machine-learning classifiers in literature.

Author (reference)	F1-score	Classifier	Dataset
Atutxa et al.^4^	0.84–0.95	RNN	Death certificates from CépiDc (France), ISTAT (Italy), and a Hungarian database^a^
Blanco et al.^6^	0.70	RNN	Osakidetza Spanish Basque public health system
Cao et al.^9^	0.68	HCAML	Internal Chinese EHR dataset
Chen et al.^10^	0.63	Longest Common Subsequence	ICD-10 National Chinese dataset
Lin et al.^14^	0.73	CNN	Tri-service General Hospital Taipei dataset with ICD-10 labels
Du et al.^11^	0.43	CNN	Multiparameter Intelligent Monitoring in Intensive Care II (MIMIC II)^b^
Duarte et al.^12^	0.65	“Combined neural network”	Cause of death autopsy reports (three-character)
Karimi et al.^13^	0.81	CNN	ICD-9 radiology reports
Koopman et al.^7^	0.94	Binary SVM classifier for 4 different codes	Australian Bureau of Statistics dataset with ICD-10 cause of deaths^c^
Pakhomov et al.^15^	0.54	Naive Bayes Classifier	Random sample of HICDA (A mayo-clinics adaptation of ICD-8) dataset
Perotte et al.^16^	0.40	Hierarchy-based SVM	Multiparameter Intelligent Monitoring in Intensive Care II (MIMIC II)^b^
Singh et al.^17^	0.86	BERT model implemented in PyTorch	Medical Information Mart for Intensive Care III (MIMIC III)
Sonabend et al.^8^	0.71	“Unsupervised knowledge integration (UNITE)”	Medical Information Mart for Intensive Care III (MIMIC III) and Partners HealthCare (PHS) Biobank^d^

*RNN* recurrent neural network, *HCAML* hierarchical convolutional attention for multi-label classification, *EHR* electronic health record, *ICD* international classification of disease, *SVM* support vector machine, *CNN* convolutional neural network, *HICDA* hospital adaptation of the international classification of diseases.

^a^Using 128,000 training data.

^b^Using the same dataset.

^c^Using 447,336 training data and only four ICD-10 codes to predict as an outcome.

^d^193,677 and 52,691 training data for six disease groups.

In our prior work, we assessed the performance of the different machine and deep learning models from literature to this dataset. We employed two vectorization methods (bag of words and word embeddings) and used support vector machines for each of the representations. We also employed several neural network architectures, from which the bidirectional gated recurrent unit (BGRU) performed best^2^. In this work, we focus on clinical usability which requires high performance, sufficient clinical granularity, and interpretability. We focussed on well-defined and frequently used three- and four-character ICD-10 codes that are clinically relevant such as atrial fibrillation (I48), acute myocardial infarction (I21) or dilated cardiomyopathy (I42.0). Since privacy-sensitive clinical data is being used, we embedded a pseudonymization algorithm in the pipeline for GDPR compliance. The main contributions of this work are: (i) addressing imbalanced data by using a binary relevance method for multiclass/multilabel classification and a combination of binary classifiers into a multilabel clinically relevant presentation, (ii) a combination of word embeddings and bi-directional gated recurrent unit neural network that encompasses neighborhood and context of words and (iii) “explainability” of the model with word coefficients and manual assessment of classification. We assessed three- and four-character performance using solely the summary paragraph of discharge letters (conclusion), adding clinical variables (age/sex) and multilabel classification, as is the case in clinical practice, and compared our proposed embedding to ELMo as a contextual embedding layer in the neural network model.

## Results

### Datasets

In total, 5548 discharge letters from in-house cardiology patients were included in the dataset with an average of 4.7 codes per letter (cardinality). The median age at discharge was 68 years (1st and 3rd quartiles [58–77]) and 36% of patients were female. For sanity check, Cohen’s Kappa was calculated for three- and four-character ICD-10 codes and was high: 0.78 (95% confidence intervals (CI) [0.72–0.84]) for four-character codes and 0.85 (95% CI [0.79–0.89]) for three-character codes. Table 2 summarizes the characteristics and an example (Box 1) is given after de-identification. Sixty-four different ICD-10 codes have at least 200 records in this dataset. The most common ICD-10 code was I25 (chronic ischemic heart disease) followed by Z95, I10, and I48 (presence of cardiac vascular implants and grafts, primary hypertension, and atrial fibrillation/flutter, respectively) with all at least 1000 individual counts. The validation dataset contained an additional 5089 discharge and procedural letters from cardiology. The most common ICD-10 code in the validation set was comparable to the training set (I25, followed by Z95, I10, I48, I50, etc.) and are depicted in the supplementary file (Supplementary Fig. 1).

**Table 2:** UMCU Cardiology dataset characteristics.

Variable	Description
Taxonomy	International Classification of Disease version 10
Language	Dutch
Number of unique records	5548
Number of unique tokens	148,726
The average number of tokens per record	936
Number of rolled-up labels (i.e., I42)	608
The average number of codes per letter	4,7
% of labels with >50 letters	8,03%
Cohen’s Kappa	4-character: 0.78, 95% CI [0.72–0.84] 3-character: 0.85, 95% CI [0.79–0.89]
Age. Median (IQs) Sex (% Female)	68 (1st: 58, 3rd: 77) years 36% Female

Box 1: An example of a Dutch discharge letter from the dataset *Bovengenoemde patiënt was opgenomen op* <*DATUM-1*> *op de* <*PERSOON-1*> *voor het specialisme Cardiologie*.

***Reden van opname*** *STEMI inferior*

***Cardiale voorgeschiedenis****. Blanco*

***Cardiovasculaire risicofactoren****: Roken(-) Diabetes(-) Hypertensie(?) Hypercholesterolemie (?)*

***Anamnese****. Om 18.30 pijn op de borst met uitstraling naar de linkerarm, zweten, misselijk. Ambulance gebeld en bij aansluiten monitor beeld van acuut onderwandinfarct*.

*AMBU overdracht. 500* *mg aspegic iv, ticagrelor 180* *mg oraal, heparine, zofran eenmalig, 3× NTG spray. HD stabiel gebleven.Medicatie bij presentatie.Geen*.

***Lichamelijk onderzoek****. Grauw, vegetatief, Halsvenen niet gestuwd. Cor s1 s2 geen souffles.Pulm schoon. Extr warm en slank*.

***Aanvullend onderzoek****. AMBU ECG: Sinusritme, STEMI inferior III)II C/vermoedelijk RCA*.

*Coronair angiografie. (…). Conclusie angio: 1-vatslijden..PCI*

***Conclusie en beleid***

*Bovengenoemde* <*LEEFTIJD-1*> *jarige man, blanco cardiale voorgeschiedenis, werd gepresenteerd vanwege een STEMI inferior waarvoor een spoed PCI werd verricht van de mid-RCA. Er bestaan geen relevante nevenletsels. Hij kon na de procedure worden overgeplaatst naar de CCU van het* <*INSTELLING-2*>*…Dank voor de snelle overname…Medicatie bij overplaatsing. Acetylsalicylzuur dispertablet 80* *mg; oraal; 1× per dag 80 milligram; <DATUM-1*>. *Ticagrelor tablet 90* *mg; oraal; 2× per dag 90 milligram; <DATUM-1*>*. Metoprolol tablet 50* *mg; oraal; 2× per dag 25 milligram; <DATUM-1*> *.Atorvastatine tablet 40* *mg (als ca-zout-3-water); oraal; 1× per dag 40 milligram; <DATUM-1*>

***Samenvatting***

*Hoofddiagnose: STEMI inferior wv PCI RCA. Geen nevenletsels. Nevendiagnoses: geen*.

*Complicaties: geen Ontslag naar: CCU* <*INSTELLING-2*>.

### Performance of models

**Fig. 3:**
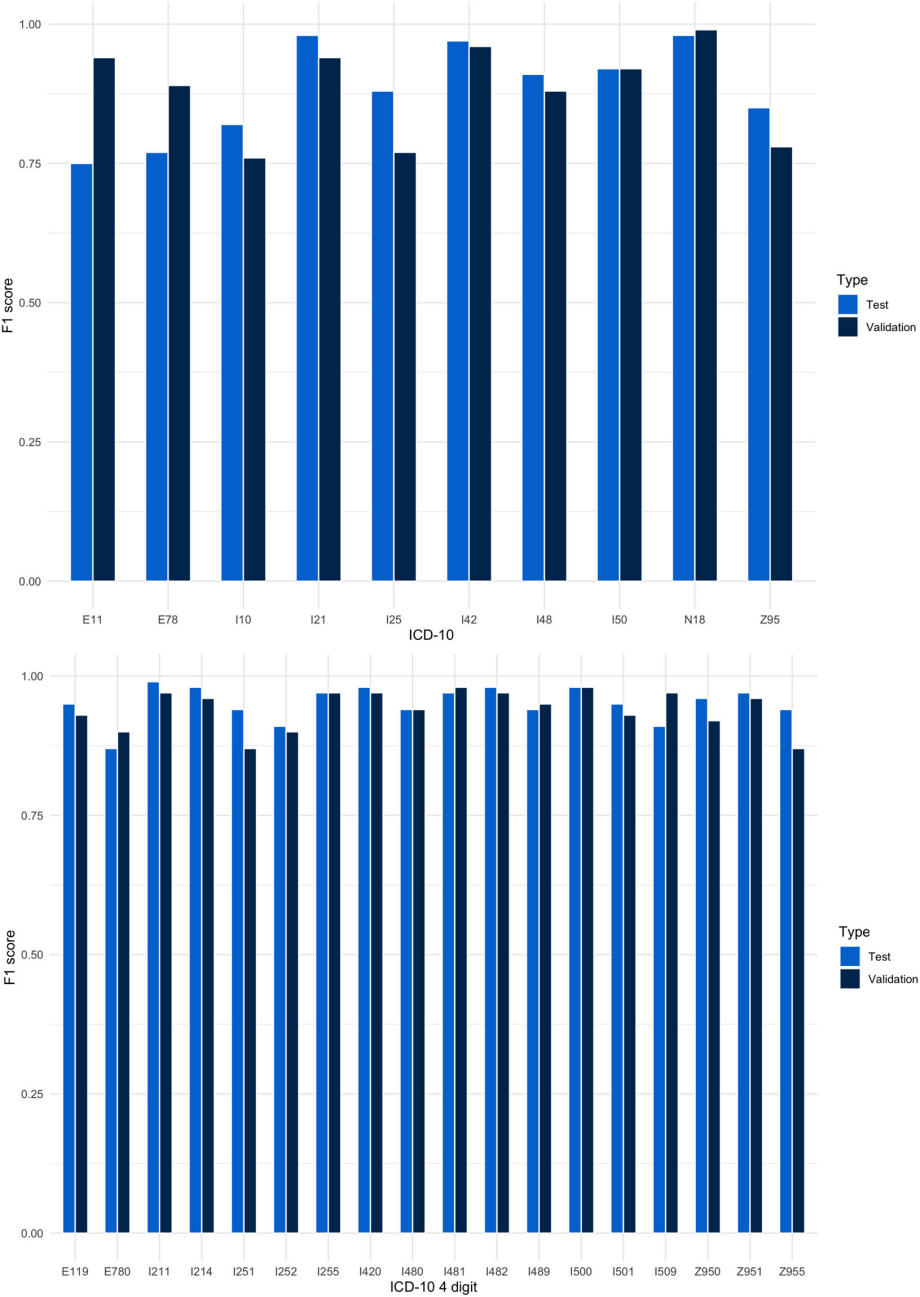
F1 scores for test and validation for three- and four-character ICD-10 codes. The model performed well in both three- and four-character codes in both the test and validation datasets.

**Fig. 4:**
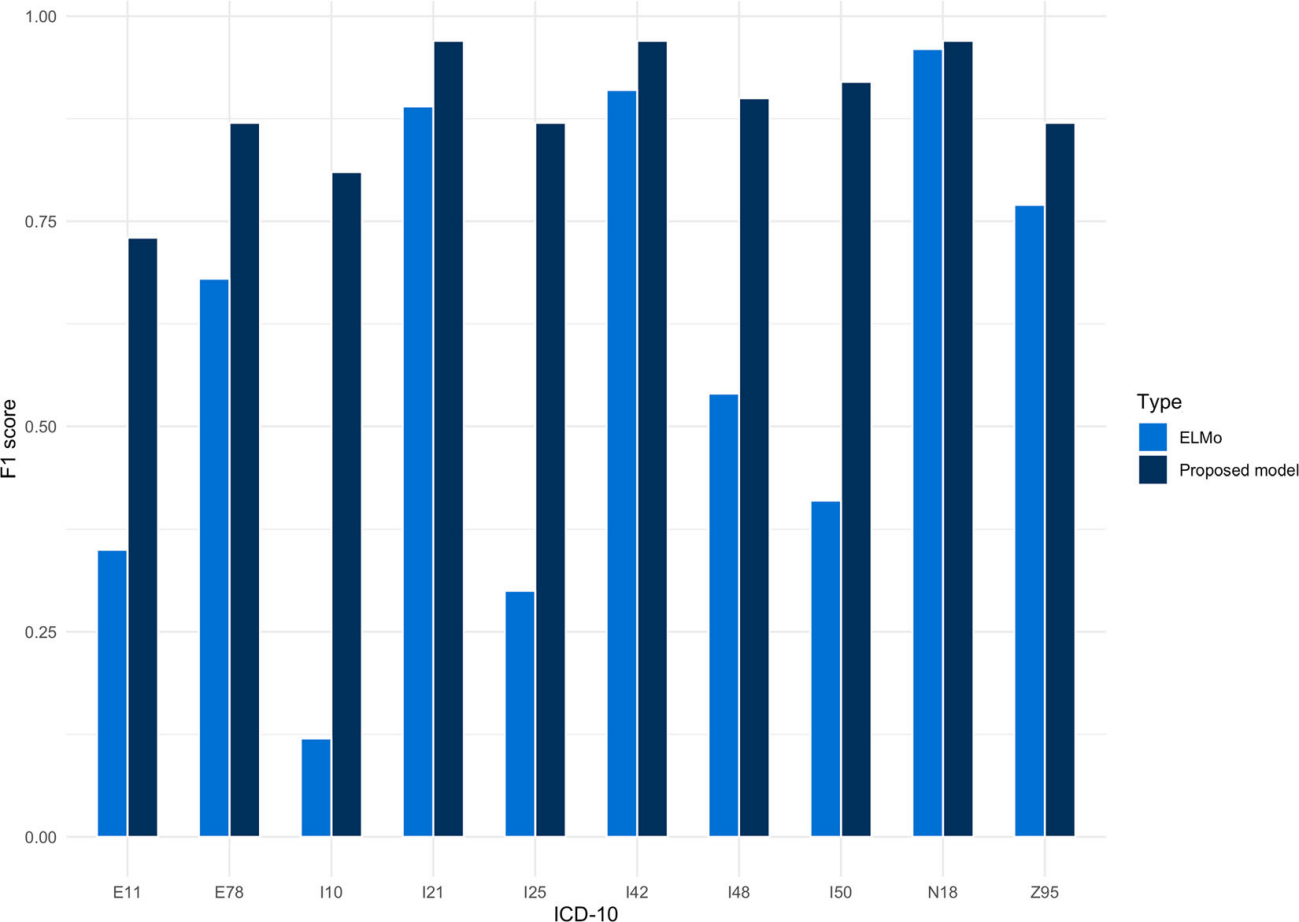
Comparison between ELMo and our proposed method. Leveraging the model by using ELMo as the embedding layer did not improve performance.

The performance in test and validation (F1-score) of our best performing model (BGRU) is summarized in Fig. 3. Overall, the performance was remarkably high for all selected ICD-10 codes in both test and validation and was optimal using the entire corpus of the discharge letters rather than using just the conclusion/summary section. Adding variables age and sex did not affect performance. Leveraging the model by using ELMo as the embedding layer did not improve performance (Fig. 4). The performance of multilabel three-character classification in the test set was 0.75 for sensitivity, 0.92 for specificity with an F1-score of 0.74 and decreased in external validation (0.72, 0.61, 0.69, respectively, Supplementary Table 5).

### Three and four-character ICD-10 labels

Table 3 contains a description of all three and four-character ICD-10 labels. Performance for main diagnosis (I21, I25, I42, I48, and I50) and cardiovascular risk factors (I10, E11, and E78) was high (Fig. 3 and Supplementary Table 2) in both test and validation. F1-scores range from 0.76 (I10) to 0.99 (N18). Performance for the four-character codes was also high, with F1 scores ranging from 0.87 (Z95.5: the presence of coronary angioplasty implant graft and I25.1: atherosclerotic heart disease of the native coronary artery) to 0.98 for I48.1 (persistent atrial fibrillation). Sensitivity in external validation ranged from 90% for the presence of cardiac and vascular implants and grafts (Z95) to 100% for cardiomyopathy (I42) (supplementary Tables 3 and 4). Specificity was lower in the validation set which would indicate false positives or over-classification by our model. For all three-character ICD-codes, these putative “false” positives were assessed. Many (83% on average) of the putative “false” positives were in fact true positives after manual review, indicating that the model had successfully identified additional cases. Of the putative “false” positives, 93% were correct for E11, 87% were correct for E78, 60% were correct for I10, and 97% correct for I21. This pattern was seen for the rest of the codes as well (Supplementary Table 7).

**Table 3:** Selected three-character and four-digit ICD-10 codes.

ICD10 code three-digit and (four-digit)	Description of codes
E11^a^ (E11.9)	Type 2 diabetes mellitus (type 2 diabetes mellitus without mention of complications)
E78^a^ (E78.0)	Disorders of lipoprotein metabolism and other lipidemias (pure hypercholesterolemia)
I10^a^	Primary hypertension
I21 (I21.1, I21.4)	Acute myocardial infarction (ST-elevation myocardial infarction and non-ST elevation myocardial infarction)
I25 (I25.1, I25.1, I25.5)	Chronic ischemic heart disease (atherosclerotic heart disease of the native coronary artery, old myocardial infarction, and ischemic cardiomyopathy)
I42 (I42.0)	Cardiomyopathy (dilated cardiomyopathy)
I48 (I48.0, I48.1, I48.2, I48.9)	Atrial fibrillation and flutter (paroxysmal atrial fibrillation, persistent atrial fibrillation, chronic atrial fibrillation, unspecified)
I50 (I50.1)	Heart failure (left ventricular failure)
N18^a^	Chronic kidney disease
Z95 (Z95.0, Z95.1, Z95.5)	Presence of cardiac and vascular implants grafts (presence of a cardiac pacemaker, presence of aortocoronary bypass graft, presence of coronary angioplasty implant and graft)

^a^Risk factor for cardiovascular disease.

### Word coefficients

To interpret the model, word coefficients have been plotted per ICD-10 code. Words that increase the prediction probability are delineated in green. For Type 2 diabetes (E11) these words are either related to the use of medication (“metformin”, “gliclazide”, “insulin”), are synonyms for E11 (“diabetes”, “mellitus”, “dmii”) or are words that co-occur with cardiovascular risk factors (“overweight” (*translation: overweight)*, “stenoses”). For hypertension (I10), the highest coefficients were reached with the synonyms and medication for hypertension as well (“hypertensie”, “amlodipine”, “valsartan”, “ht”). This pattern can be seen for all ICD-10 codes. The words “Blanco”, “normale” and “nee” all have negative coefficients which illustrate the negative effect of these words in the ICD-10 codes E11, E78, I10, I21, and Z95. The coefficients of all ICD-10 codes are visible in the Supplementary Files.

### Manual qualitative assessment of classification

For qualitative assessment of over-, under-, and improved classifications all three-character ICD-10 codes were investigated manually by a clinical doctor. The model performed remarkably well in predicting ICD-10 codes of patients in case medication use indicated specific diagnoses. For E11 (type 2 diabetes) for example, in case metformin or gliclazide was prescribed, the model accurately identified them whereas the medical coders missed them in the validation set. The model seemed to overestimate the probability of type 2 diabetes when “type ii” was used in another context (type 2 ischemia or type 2 atrial septal defect). This detection of prescribed medication in the text was also present for hypertension (I10) and dyslipidemias (E78). The detection of medication, however, also led to overclassification, since some prescribed drugs (amlodipine, perindopril, or rosuvastatin) are often also prescribed as a means of treatment or primary/secondary prevention in other diseases than hypertension, for example in heart failure or ischemic heart disease. In the case of acute myocardial infarction (I21), the model accurately identified procedures for which acute ischemia was an indication (STEMI and non-STEMI). Our model seemed to struggle with shorter ambiguous procedural letters. In the case of I50 (heart failure) relatively short discharge letters (e.g., for device implantation) may include an abbreviation of cardiac decompensation *(“decomp cor”)* but was missed by our model. As expected, if more words were used to describe the patient’s condition (*“CRT-D replacement for non-reversible perfusion defects that led to a dilated and poorly functioning asynchronous LV”)* the model did accurately predict the ICD-10 class. Overclassification was present in case of other reasons for decompensation than cardiac (pulmonary, hepatic, or renal), or in case cardiomyopathy was not yet diagnosed but the discharged patient was still undergoing the work-up. Supplementary Table 8 contains a description of all three-character ICD-10 codes and their qualitative assessments.

## Discussion

We created a deep learning pipeline for automatic multilabel ICD-10 classification in the free medical text using Dutch cardiology discharge letters. Given the sensitive nature of these data, we included a de-identification step^22^.

Prior work on NLP in cardiology was focused on specific relevant indicators such as hypertension, algorithms to identify Framingham heart failure signs and symptoms, or identification of cardiovascular risk factors and outcomes^23^. The use of recurrent neural networks (RNN) for cardiovascular diagnoses, risk factors, and complications, however, remained relatively uncharted. Partially, this is due to the rather low performance of some models limiting clinical usefulness^7,9–16^. Recent methodological developments in neural networks lead to high performing models, but they rely on limiting the number of codes (four) to predict or require huge datasets of up to 128,000 training data points (Table 1)^4,7^. Limited performance of some models, the necessity of gigantic datasets for (pre-)training, and lack of interpretability withhold them from replacing or aiding a human coder.

In this work, we used a deep neural network and focussed on clinical usefulness with both single and multilabel prediction in a relatively small dataset of 5,548 clinical discharge notes. We extracted frequently used, well defined, and clinically relevant three- and four-digit ICD-10 codes^5^. These three-character codes still have enough granularity to include relevant diagnoses such as atrial fibrillation (I48) or acute myocardial infarction (I21). Next, we assessed and improved an already potent type of RNN (BGRU) by using semi-structured parts of the text, by adding clinical variables (age and sex), and by adding an ELMo embedding layer. We then sought to explain our model using word coefficients and a manual review of misclassifications. Even though our dataset focussed on cardiology, the pipeline is generalizable and may be trained with data from any other specialty.

A comparison of several state-of-the-art RNN ICD coding systems reported that classification performance is higher for ICD chapters than rolled-up codes. The previously reported F1-scores of ICD-10 chapters for this dataset were around 50–60% at best and limited to 20–30% for rolled-up, more terminal codes^2^. BGRU has been promising for the classification of medical text and prior experiments advocate either reducing granularity or increasing training data to improve performance^2,4,6^. In addition, the use of co-occurrences (association rule mining) for the initialization weights also positively impacted performance^12^. Unfortunately, in most settings training data are limited. Therefore, we tried reducing the granularity of our dataset whilst remaining clinically relevant without reducing the label-set size. By doing so, our pipeline reached F1 scores for rolled-up codes of 97%. Using the entire corpus of text rather than semi-structured parts also improved classification performance, especially for conventional risk factors such as diabetes and hypertension that are seldomly mentioned in the summary paragraph of discharge letters. By building on prior work and using BGRU which is computationally less expensive, our reported performance is substantially higher than previously seen in smaller datasets, making it a useful and scalable tool for administrative and research support^2,4,12^. We argue that this is caused by the high-quality of the selected training data, our preprocessing pipeline, and the binary classification method together with a potent BGRU. Contextual word embeddings (ELMo and BERT) have shown substantial performance improvements in many NLP tasks^6,18,19^. Recently, Blanco et al. assessed the performance of a BGRU combined with ELMo, showing an improvement in model performance. Their trained language was in Spanish which in terms of NLP is under strong growth and therefore they were able to train their embedding sets on the strong Spanish Billion Word Corpus^6^. In this regard, however, the lack of large Dutch (medical) language models for embeddings poses an important challenge. This is especially understandable as in our case privacy sensitive information in the medical field may be compromised if these language models are published online^21^. Interestingly, ELMo did not positively affect our results which may be due to a variety of reasons. First, our pipeline was already optimized for this specific task of medical ICD-10 labeling and included word-embedding in the first layer of the BGRU, performing quite well with a binary relevance method. Next, given the fact that our model is trained and validated in a specific field of expertise (cardiology), there is little word ambiguity to be expected (the case when contextual word embeddings would be most beneficial). Third, the ELMo pipeline may still be suboptimal and have room for improvement for this task. Using language-specific pre-trained embeddings in the field of medicine, multi-language support, or by trying meta-embeddings as proposed by Blanco may further improve the performance of these pipelines^6,24^. A recently published standardized benchmarking by Peng et al.^25^ evaluated BERT and ELMo on ten datasets, showing substantially better performance using pre-trained BERT models than other state-of-the-art models. Sing and colleagues implemented BERT as well on de-identified data from the MIMIC-III dataset (58.000 admissions). They demonstrated that with fine-tuning based transfer learning of a pretrained bidirectional transformer language model, very high overall performances can be reached for both top 10 and top 50 ICD-10 codes. They advocate working on interpretability for models’ prediction and further deployment to more coding systems (e.g., CPT and SNOMED)^17^.

An important consideration is model interpretability. State-of-the-art deep learning models are challenging to grasp with no specialized knowledge in neural networks, and practice has shown that the easier the model, the wider its acceptance. There has been a significant increase in the use of machine learning methods but a notable proportion of works still use relatively simple methods: shallow classifiers, or combined with rule-based methods for higher interpretability^23^. Interpretable results however may provide experts with supporting evidence when confronted with coding decisions^4^. We, therefore, attempted to provide insight into the model by using word coefficients and manual assessment of classifications. These results illustrate that synonyms of ICD-10 diagnoses or medication specifically prescribed for these diseases have the highest positive probabilities. Negative words (negation), such as “normal*”* or “no” decrease the probability of ICD-10 diagnoses, more noticeably for cardiovascular risk factors. Interestingly, in a recent study published by Lin et al.^20^, their results also suggest that BERT subsumes domain adaptation for negation detection and further fine-tuning on specific corpora does not lead to much overfitting.

Most ICD-10 codes are used rarely in clinical practice, while a small number of diagnoses comprise the majority of patients seen in cardiology clinics^3,5^. To aid administrative support, our focus was directed towards multilabel classification and we argue that the model is interpretable and its performance is high enough to aid medical coders. From a clinical perspective, the high single label performance allows for patient identification in EHRs by using only the clinical discharge letters as a first step towards building research cohorts of interest. Less frequent ICD-10 codes, for rare diagnoses for instance, still require datasets large enough for machine learning and deep learning algorithms to perform well in ICD-10 classification^2^. For these diagnoses, rule-based methods may be a more viable option, given that the terms in the text follow regular patterns and the task is limited to single-label classification^4^. To accurately capture rare diagnoses, other more structured parts of the EHR may be useful such as laboratory results. A well-performing example is a simple classification algorithm for the identification of patients with systemic sclerosis in the EHR by using positive antinuclear antibody titer thresholds^26^.

An automated coding system that combines simple classifiers with machine learning models is not new, as they have been successfully implemented in 2006 at the Mayo Clinic and resulted in an 80% reduction of staff engaged in manual coding^15^. More recently, a similar system for veterinary EHRs (VetTag) was built, which classified veterinary clinical notes with diagnosis codes. Authors argue that processing these clinical notes has a tremendous impact on (veterinary) clinical data sciences^27^. Nonetheless, these promising results have not led to the widespread use of automatic coding systems for discharge letters^23^. It is clear that human coders can benefit by reviewing suggested ICD-10 codes rather than reading all discharge letters and translating them to proper ICD-10 codes^15^. Saved time can then be used to dive deeply into the correct terminal and detailed coding or additional structuring of data, leading to better research infrastructure. However, there are two long-term concerns: the first is the actual implementation of these algorithms into the software. Implementation is more than solely installing an automation pipeline. It requires new software that is embedded in existing workflows and prolonged maintenance. The second is the improvement of technology for more complex and less frequent ICD-10 codes with high accuracy, which would require larger datasets and feedback algorithms. We underline the importance of further efforts to focus on implementation, rather than solely focusing on methodological fine-tuning as suggested by Singh et al.^17^.

Our proposed model may be limited by the quality of the data. Even though they were coded by an experienced medical coder, given the character of the dataset it is prone to have human error. As this work involves privacy-sensitive data, we are restricted by the Dutch version of the European GDPR (AVG) which inhibits us from using external Dutch datasets. Nonetheless, within this small country and the fact that medical staff rotate we do not believe this poses a major limitation to the validation. Future studies may improve this model by using contextual word embeddings pre-trained on Dutch medical corpora, assess performance in other datasets as well as the use of other coding systems.

We propose a novel automated ICD-10 classifier BGRU pipeline with a de-identification step. Interpretation of the BGRU pipeline is made possible by using word coefficients. Because of its high performance, this pipeline can be useful to decrease the administrative burden of classifying discharge diagnoses and may serve a scaffold for reimbursement and research applications.

## Methods

### Medical ethical regulations and GDPR

This study was exempt from medical ethical regulations by the Medical Ethical Committee of the University Medical Center Utrecht (UMCU) (No. 18-446). A data management plan was created and reviewed by the privacy security board to meet institutional and national requirements in the Netherlands for GDPR compliance.

### Dataset

Discharge letters were retrieved from the EHRs in the University Medical Centre Utrecht (UMCU) and were available from the start of the EHR on 08-09-2013 until data extraction on 30-06-2018, written by a total of 84 different medical doctors. All letters were manually classified with multilabel/multiclass ICD-10 codes by an experienced medical coder that works solely in the field of cardiology. The discharge letters were matched to the corresponding ICD-10 classification by using patient ID and dates of admission/discharge from within the UMCU Research Data Platform. We removed ICD-10 codes with less than 50 observations^28^. Since the reliability of terminal codes is poor, simplification of ICD-10 codes is important to receive a valid image of health care reality^5^. The selection of specific ICD codes was based on availability and clinical usability (sufficient granularity) of higher-level rolled-up codes (e.g., I42 (cardiomyopathy) rather than I42.3 (endomyocardial (eosinophilic) disease)). The 10 selected codes account for six main diagnoses (acute myocardial infarction, chronic ischemic heart disease, cardiomyopathy, atrial fibrillation/flutter, heart failure and presence of cardiovascular implant grafts) and four cardiovascular risk factors (type 2 diabetes, hyper/dyslipidemia, primary hypertension, chronic kidney disease). To not oversimplify the task, from these 10 selected codes, further four-character ICD-10 codes (e.g., I48.0 (paroxysmal atrial fibrillation) rather than I48) were also considered to assess performance for very granular labels with at least 100 appearances in both the training and validation set. The ICD-10 codes are depicted in Table 3. Dataset quality for both three- and four-character ICD-10 codes were manually assessed using an adaptation of Cohens Kappa previously described and used for ICD-10 codes (AS)^5^. Hundred clinical discharge notes were randomly selected, stripped from patient-IDs, and reclassified by a medical coder (D.K.) that was blinded to the correct codes.

### Validation

To assess the performance of the model in a new dataset, a nonoverlapping temporal validation dataset was created consisting of letters and ICD-10 codes. This validation set contains new clinical discharge and procedural letters written by 46 different medical doctors. Given GDPR restrictions and the nature of this privacy-sensitive work, extracting letters from other hospitals was not possible. However, because clinical staff in the Netherlands rotate from hospitals within the country frequently, the letters were written by other clinicians and teams. In addition, sentence structures, as well as diagnosis coding structures, are interchangeable in hospitals. Therefore, this temporal dataset was deemed fit for external validation. For this set, clinical letters from 01-07-2018 until 04-09-2019 were included. Because the dataset is solely constructed on discharge letters and ICD-10 codes, the pipeline is not EHR system or vendor-specific and may be interoperable.

### Machine-learning pipeline for ICD-10 classification

**Fig. 5:**
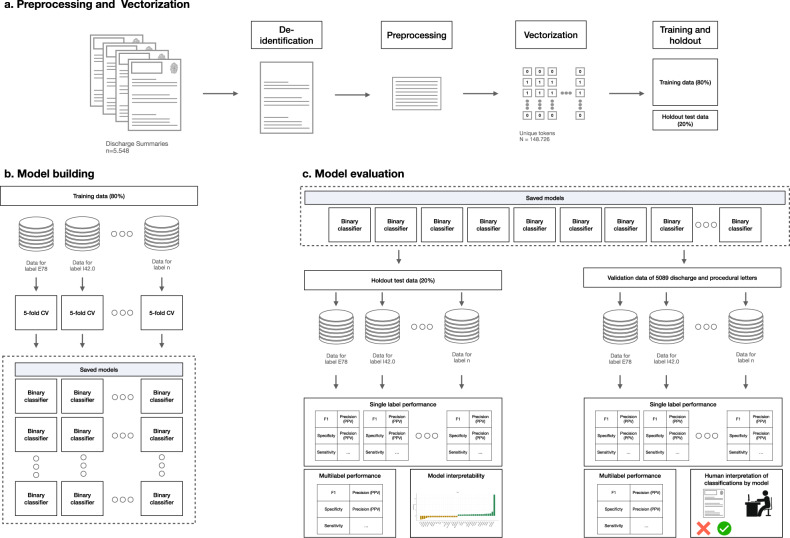
Summary of training, validation, and model interpretation pipeline. Data were preprocessed, vectorized, and split into a training (80%) and holdout (20%) set as shown in panel (**a**). Binary classifiers were trained in panel (**b**) and the model was evaluated in panel (**c**). Model interpretability was provided by using word coefficients and human interpretation of misclassification.

The pipeline is summarized in Fig. 5. Before feeding data into the different machine learning or deep learning algorithms, we first applied the following steps:

(i) We de-identified the letters using *DEDUCE*^22^.
(ii) We preprocessed the text (trimmed whitespaces, numbers and converted all characters to lowercase) using the *tm* and *tidytext* packages in *R*^29^.

To transform the text into data a machine can understand (text representation), the output of our preprocessed text was then vectorized using word embedding. This method allows representing words in such a way that it captures meanings, semantic relationships, and context that words are used in. It is a dense feature representation in a low dimensional vector and has been proven to be a robust solution for most NLP issues. Word embedding is also the first layer in a neural network (NN) based classifier. After *k*-fold cross-validation (*k* = *5)* we implemented a BGRU NN.

### BGRU neural network

**Fig. 6:**
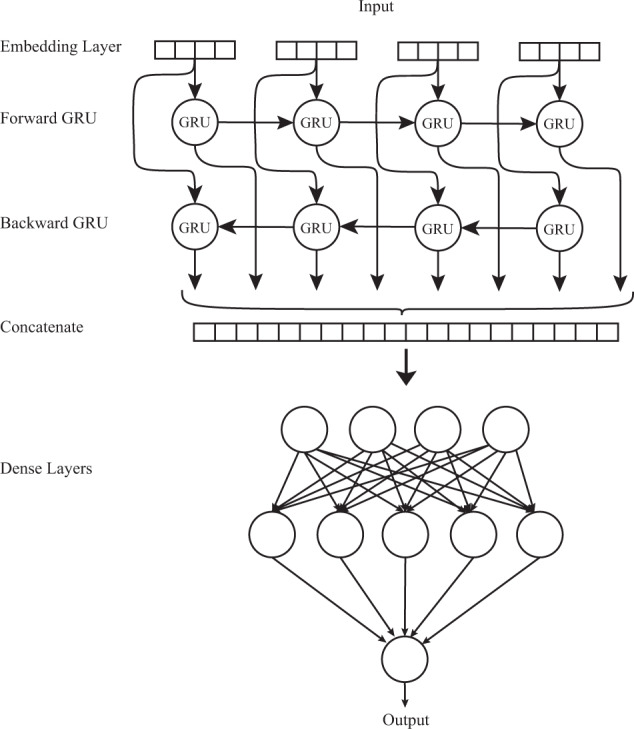
Bidirectional Gated Recurrent Unit Neural Network (BGRU). The general architecture of a BGRU is illustrated here. It can model sequential data and by using recurrent connections, information can cycle inside these networks for an arbitrarily long time.

The general architecture of a BGRU model is shown in Fig. 6. In this model, the input layer is the text from discharge letters and the output layer is the ICD-10 label. The model uses deep RNN in its hidden layers, called gated recurrent units (GRUs). GRU is a type of RNN that can model sequential data. The GRU network receives an input at each timestep, updates its hidden state, and makes a prediction. By using recurrent connections, information can cycle inside these networks for an arbitrarily long time. However, RNNs are known to have difficulties learning the interactions between distant words because of long-range dependencies. This problem is known as the vanishing gradient problem. Extensions for NNs, such as long-short term memory (LSTM) and GRU were specifically designed to combat this issue through a gating mechanism. Using GRUs also leads to a reduced number of parameters, faster convergence, and a more generalizable model in comparison to other methods^12^.

We used the *Keras* library to implement the BGRU model for automated ICD-10 coding^30^. Vector dimensionality was set to 300, windows size to five and we discarded words that only appeared once in the training set. We experimented with the model directly on the word sequence of all the discharge letters. As in previous studies on textual data, the fact that our data contains long texts creates a challenge for preserving the gradient across thousands of words. Therefore, we used dropout layers to mask the network units randomly during the training^31^. We set the number of hidden units in the RNN layers at 100. Dropout and recurrent dropout were added to avoid overfitting, both at a 0.2 rate. On the output of the recurrent layer, a fully connected NN (two dense layers) was applied for the classification of the ICD-10 codes. The hidden dense layer contains 128 units and uses the *relu* activation function, and the output layer uses a softmax function to determine if the ICD code should be assigned to the letter.

### Contextual word embeddings

A dense NN using word vectors from contextual embeddings based on ELMo has been used for the comparison study^19^. These word vectors are learned functions of the internal states of a deep bidirectional language model trained on our original dataset. In this representation, the vector obtained for each word depends on the entire context in which it is used. Using a bidirectional LSTM, instead of a fixed embedding for each word, ELMo looks at the entire sentence before assigning each word an embedding (Supplementary Fig. 2).

### Assessment of performance and experiments

We investigated performance by randomly splitting the dataset between a training (0.80) and testing (0.20) set. The model was then again evaluated in external validation. Sensitivity (recall), specificity, positive predictive value (PPV, precision), negative predictive value (NPV), and F1-score (a harmonic mean between sensitivity and positive predictive value) were calculated. We performed four experiments with different input variables: (I) using only the summary paragraph parts of discharge letters (conclusion), (II) using the entire corpus of discharge letters, (III) using the entire corpus of discharge letter and adding the variables age and sex, and (IV) multilabel classification of experiment III. For an administrative support tool, it is important to suggest the right diagnoses, ranked by the prediction probabilities. For multilabel assessment, we considered every ICD label above a probability threshold as a positive. We assigned this threshold in such a way that the label cardinality for the test set is similar to the label cardinality in the training set. When performance discrepancies were present, a clinical doctor (A.S.) manually assessed these errors in a descriptive manner. False positives were either all manually assessed, or a subset of 100 letters in case of >100 putative false positives.

### Reporting summary

Further information on research design is available in the Nature Research Reporting Summary linked to this article.

## Supplementary information

Supplementary Information

Reporting Summary

## Data Availability

The dataset is not available due to patient privacy restrictions. However, the model may be shared with qualified researchers from academic or university institutions upon request via the corresponding author.

## Code availability

The code used in this study can be found at GitHub: https://github.com/bagheria/cardio-icd-assignment.
