# Supplementary material for: Automatic multilabel detection of ICD10 codes in Dutch cardiology discharge letters using neural networks
Source: NPJ Digit Med. 2021 Feb 26;4:37. doi: 10.1038/s41746-021-00404-9 (PMC7910461; doi:10.1038/s41746-021-00404-9)
Supplement: Supplementary file 1 — Supplementary Information [file 41746_2021_404_MOESM1_ESM.pdf]

Supplementary file: Automatic multilabel detection of ICD10 codes in Dutch cardiology discharge letters using neural networks

Supplementary figure 1

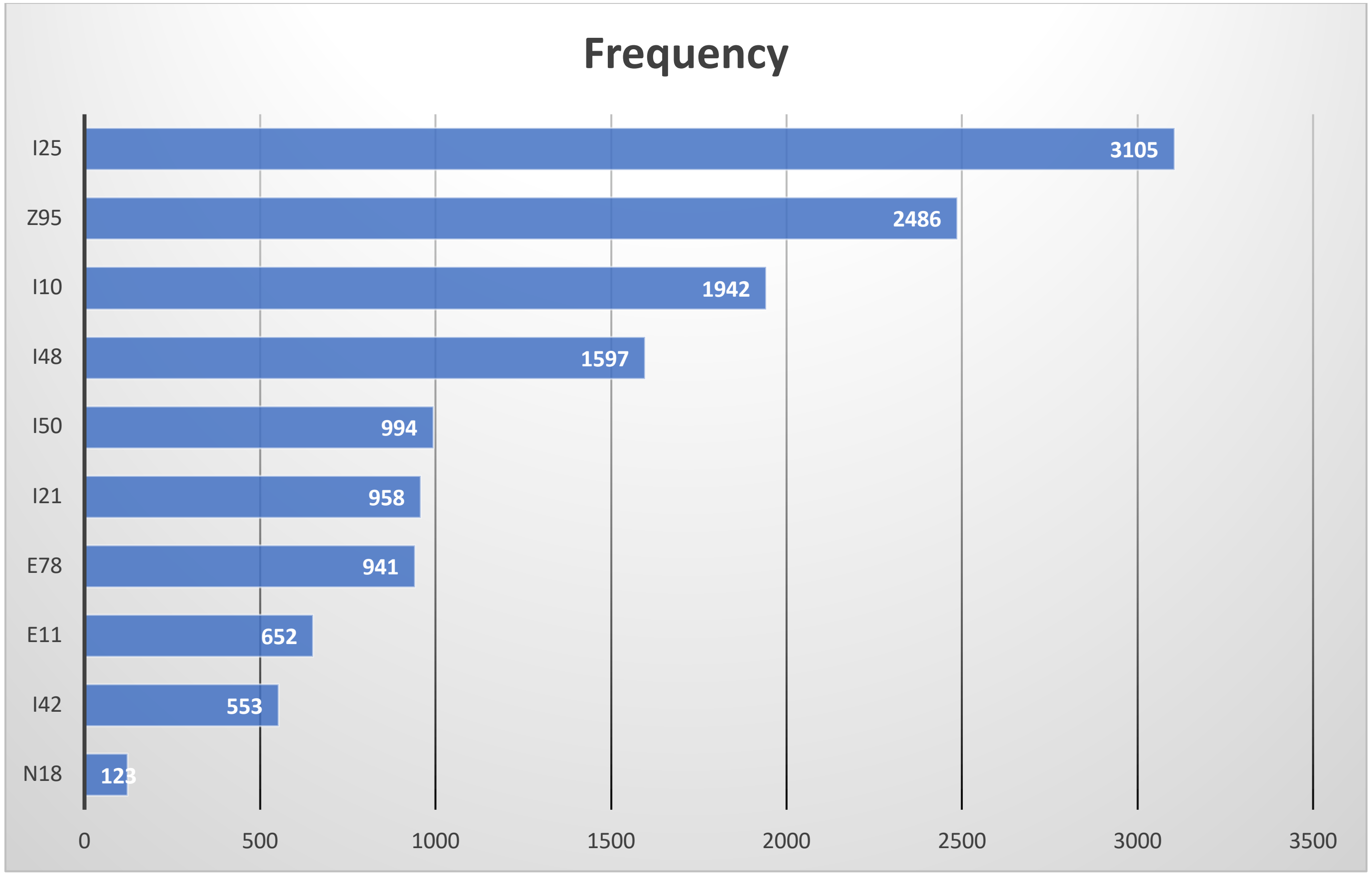

Supplementary Table 2: Results for rolled-up codes in text

Cardiovascular disease classification using only specific structured parts of discharge letters (conclusion/summary)

	E11	E78	I10	I21	I25	I42	I48	I50	N18	Z95
**Sensitivity**	0,67	0,69	0,78	0,98	0,88	0,99	0,95	0,95	0,99	0,86
**Specificity**	0,96	0,58	0,59	0,81	0,63	0,46	0,73	0,69	0,14	0,48
**Pos Pred Value**	0,73	0,83	0,70	0,97	0,81	0,92	0,84	0,88	0,94	0,76
**Neg Pred Value**	0,95	0,39	0,68	0,87	0,73	0,85	0,90	0,85	0,60	0,64
**F1**	0,70	0,75	0,74	0,97	0,84	0,95	0,89	0,91	0,96	0,81

Cardiovascular disease classification using complete discharge letters

	E11	E78	I10	I21	I25	I42	I48	I50	N18	Z95
**Sensitivity**	0.71	0.70	0.84	0.99	0.88	0.98	0.93	0.93	1.00	0.87
**Specificity**	0.97	0.64	0.73	0.80	0.76	0.75	0.82	0.76	0.45	0.65
**Pos Pred Value**	0.79	0.85	0.79	0.97	0.87	0.96	0.89	0.90	0.97	0.83
**Neg Pred Value**	0.95	0.42	0.79	0.91	0.78	0.88	0.87	0.82	0.89	0.73
**F1**	0.75	0.77	0.82	0.98	0.88	0.97	0.91	0.92	0.98	0.85

Cardiovascular disease classification using complete discharge letters and variables age and sex

	E11	E78	I10	I21	I25	I42	I48	I50	N18	Z95
**Sensitivity**	0,70	0,70	0,82	0,98	0,88	0,99	0,94	0,92	0,99	0,90
**Specificity**	0,97	0,63	0,74	0,83	0,74	0,74	0,79	0,78	0,42	0,65
**Pos Pred Value**	0,77	0,85	0,80	0,97	0,86	0,96	0,87	0,91	0,96	0,84
**Neg Pred Value**	0,95	0,42	0,77	0,86	0,77	0,90	0,89	0,81	0,78	0,76
**F1**	0,73	0,77	0,81	0,97	0,87	0,97	0,90	0,92	0,97	0,86

Supplementary Table 3: results for rolled-up codes in external validation

	E11	E78	I10	I21	I25	I42	I48	I50	N18	Z95
**Sensitivity**	0.98	0.99	0.91	0.97	0.94	1.00	0.99	0.98	1.00	0.90
**Specificity**	0.30	0.03	0.30	0.61	0.57	0.36	0.44	0.29	0.13	0.39
**Pos Pred Value**	0.90	0.80	0.65	0.91	0.65	0.92	0.79	0.87	0.98	0.69
**Neg Pred Value**	0.73	0.57	0.71	0.83	0.92	0.91	0.94	0.77	0.56	0.73
**F1**	0.94	0.89	0.76	0.94	0.77	0.96	0.88	0.92	0.99	0.78

Supplementary Table 4: results for four-character codes in external validation

Metric	E780	I252	I509	I251	E119	I480	Z950	Z955	I501	I489	Z951	I420	I255	I481	I214	I211	I500	I482
**Sensitivity**	1,00	0,99	0,99	0,96	0,99	0,99	0,98	0,99	1,00	1,00	1,00	1,00	1,00	1,00	1,00	1,00	1,00	1,00
**Specificity**	0,02	0,10	0,04	0,69	0,22	0,28	0,25	0,07	0,04	0,08	0,25	0,36	0,07	0,30	0,22	0,11	0,07	0,11
**Pos Pred Value**	0,82	0,83	0,95	0,79	0,88	0,89	0,87	0,78	0,88	0,90	0,93	0,95	0,95	0,96	0,92	0,95	0,96	0,95
**Neg Pred Value**	0,62	0,68	0,23	0,93	0,81	0,89	0,74	0,62	0,55	0,69	0,88	0,91	0,86	0,83	0,85	0,87	0,93	0,78
**F1**	0,90	0,90	0,97	0,87	0,93	0,94	0,92	0,87	0,93	0,95	0,96	0,97	0,97	0,98	0,96	0,97	0,98	0,97

Supplementary Table 5: multilabel classification performance

Metric	Proposed BGRU (test)	ELMo	Proposed BGRU (validation)
**Sensitivity**	0.75	0.78	0.72
**Specificity**	0.92	0.61	0.61
**Pos Pred Value**	0.74	0.40	0.65
**Neg Pred Value**	0.91	0.88	0.87
**F1**	0.74	0.56	0.69
**True cardinality**	2.57	2.57	2.53
**Prediction cardinality**	2.39	2.39	2.17

Supplementary Table 7: Manual review of true positives

Metric	E11	E78	I10	I21	I25	I42	I48	I50	N18	Z95
**Actual true positives (correct/putative false)**	93% (66/71)	87% (17/20)	61% (100 samples)	97% (109/112)	81% (91/113)	83% (15/18)	70% (28/40)	97% (63/65)	83% (10/12)	82% (100 samples)

Supplementary Table 8: Qualitative assessment of reasons for over-, under-, and improved classification of ICD-10 codes.

ICD-10 code	Reasons for over-classification	Reasons for under-classification	Reasons for improved classification
E11 (Type 2 DM)	-Ambiguity in "Type ii" (i.e. for type ii atrial septal defect, or type ii ischemia, or type 2 AV-block)<br>-The model misinterpreting negation (*"Geen DM"*)	-Ambiguity in typing (i.e. dmii)<br>-Procedural letters that did not describe history (for which history is derived by using other letters)	-Medication use (metformin, gliclazide, insulin)
E78 (Dyslipidemia)	-Family history mentioned, but not specifically for dyslipidemia's	-Procedural letters that did not describe history (for which history is derived by using other letters)	-Medication use (rosuvastatin, simvastatin)
I10 (Hypertension)	-Overlapping indications for medication prescriptions (i.e. perindopril for hypertension or heart failure)<br>-Procedural letters that measure bloopressure and describe the effect of verapamil<br>-Pulmonary hypertension	-Procedural letters that did not describe history (for which history is derived by using other letters)<br>-Too short description of hypertension in history (one word without context)	-Medication use (amlodipine, perindopril)
I21 (Acute myocardial infarction)	-(un)stable angina for which PCI was performed	-Short procedural letters	-Additional identification of nstemi and stemi for which PCI was performed in discharge letters
I25 (Chronic ischemic heart disease)	-Ischemia in differential diagnosis but not conclusive (passed thrombus, microvascular)<br>-Ischemia other than cardiac due to aortic aneurysm (renal, femoral)<br>-Coronary artery calcification present, but not significant (or clinically relevant)	-Ambiguous spelling of ischemia<br>-Short procedural letters for PCI	-Identification of stable or unstable angina<br>-Medication use of sublingual NTG without explicit mention of ischemic heart disease
I42 (Cardiomyopathy)	- ischemic cardiomyopathy (in essence excluded in I42)<br>- Still in work-up for I42, not yet diagnosed<br>- Ambigious, ischemia but hypertrophic or dilated cardiomyopathy in differential diagnosis	- Ambiguous diagnosis (decompensation cordis) without explicit mention of work-up for and diagnosis of cardiomyopathy<br>- Abbreviated diagnoses (dcm eci wv hmii)	- Identification of decompensation cordis with explicit mention of work-up without mention of diagnosis (non-ischemic decompensation cordis)<br>- myocarditis leading to heart failure
I48 (Atrial fibrillation/flutter)	-Electrocardioversions for ventricular tachycardia rather than for atrial fibrillation/flutter<br>-Supraventricular tachycardia in differential diagnosis, but not actually diagnosed<br>-Acenocoumarol for deep venous thrombosis rather for atrial fibrillation/flutter<br>-Supraventricular tachycardia in differential diagnosis but not actually diagnosed.	-Actual diagnosis not described in discharge letter, but treatment (cardioversion) and complication (TIA) were mentioned.<br>-Actual diagnosis missing in discharge letter, but treatment strategy mentioned (rate/rhythm control)	-Correct identification of subtle/ambigious typing ("af" or "paf")
I50 (Heart failure)	- Decompensated for other reason than cardiac (pulmonary, or hepatic)	- acute decompensation (asthma cardiale)<br>- abbreviations (e.g.: deco cor, decoco)<br>- short reports	- Admissions because of complications of heart failure (cardiac arrest due to heart failure)<br>- Procedures that do not explicitly mention "heart failure" but it can be deduced (CRT-D replacement for non-reversible perfusion defect, dilated and poorly functioning LV)
N18 (Chronic Kidney Disease)	- Consultation by nephrologist but no explicit mention of chronic kidney failure	- Procedural letters that did not describe history (for which history is derived by using other letters)<br>- Kidney transplantation in history	- patients on (peritoneal) dialysis
Z95 (Implantation of graft or device)	- Indication for device, graft or stent implantation is discussed, but planned for the future<br>- Clinical indication for device, graft, or stent, but not chosen due to other clinical reasons (e.g. bad prognosis)	- Short reports for device lead replacement<br>- Short mention of device in history, but admission for other main diagnosis problem	- Subtle mention of Mitraclip in history<br>- PCI with stenting or ICD/CRT-D implantation<br>- Function of prior CABG described in new report with CAG without mention of CABG in history

Supplementary Figure 2: Architecture of ELMo based Neural Network

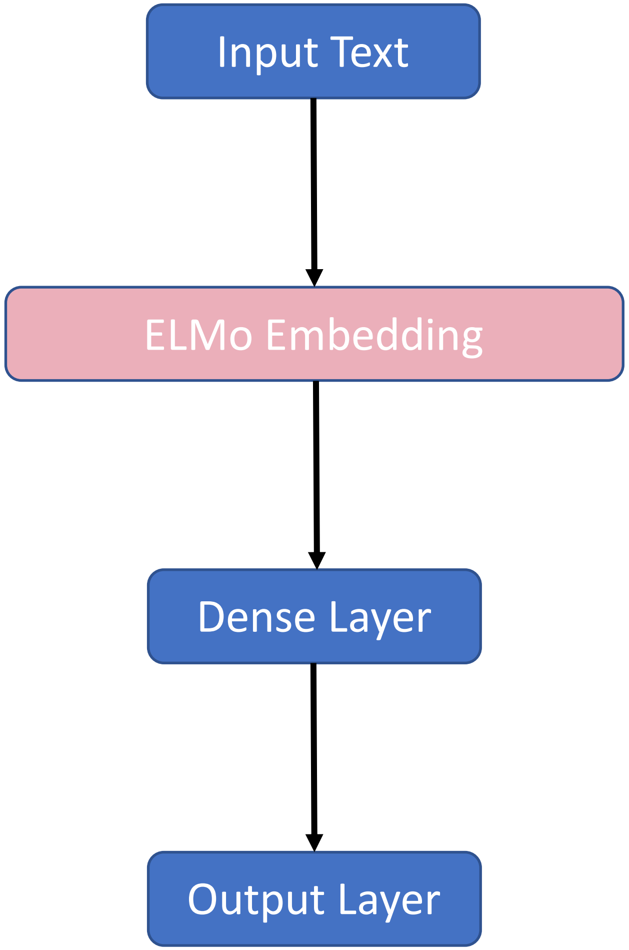

Word coefficients per three-digit ICD-10 code (pages 9-18/18)

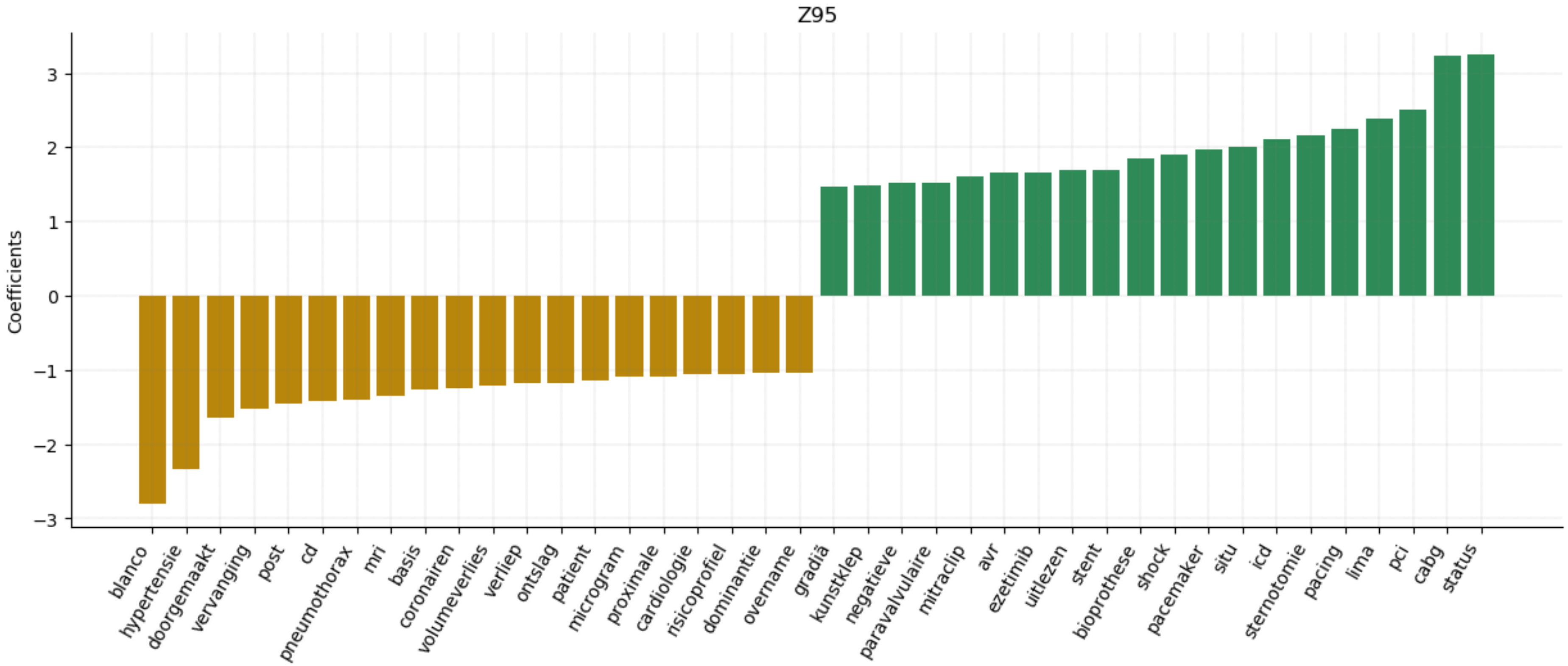
Z95
Coefficients
3
2
1
0
−1
−2
−3
blanco
hypertensie
doorgemaakt
vervanging
post
cd
pneumothorax
mri
basis
coronairen
volumeverlies
verliep
ontslag
patient
microgram
proximale
cardiologie
risicoprofiel
dominantie
overname
gradiä
kunstklep
negatieve
paravalvulaire
mitraclip
avr
ezetimib
uitlezen
stent
bioprothese
shock
pacemaker
situ
icd
sternotomie
pacing
lima
pci
cabg
status

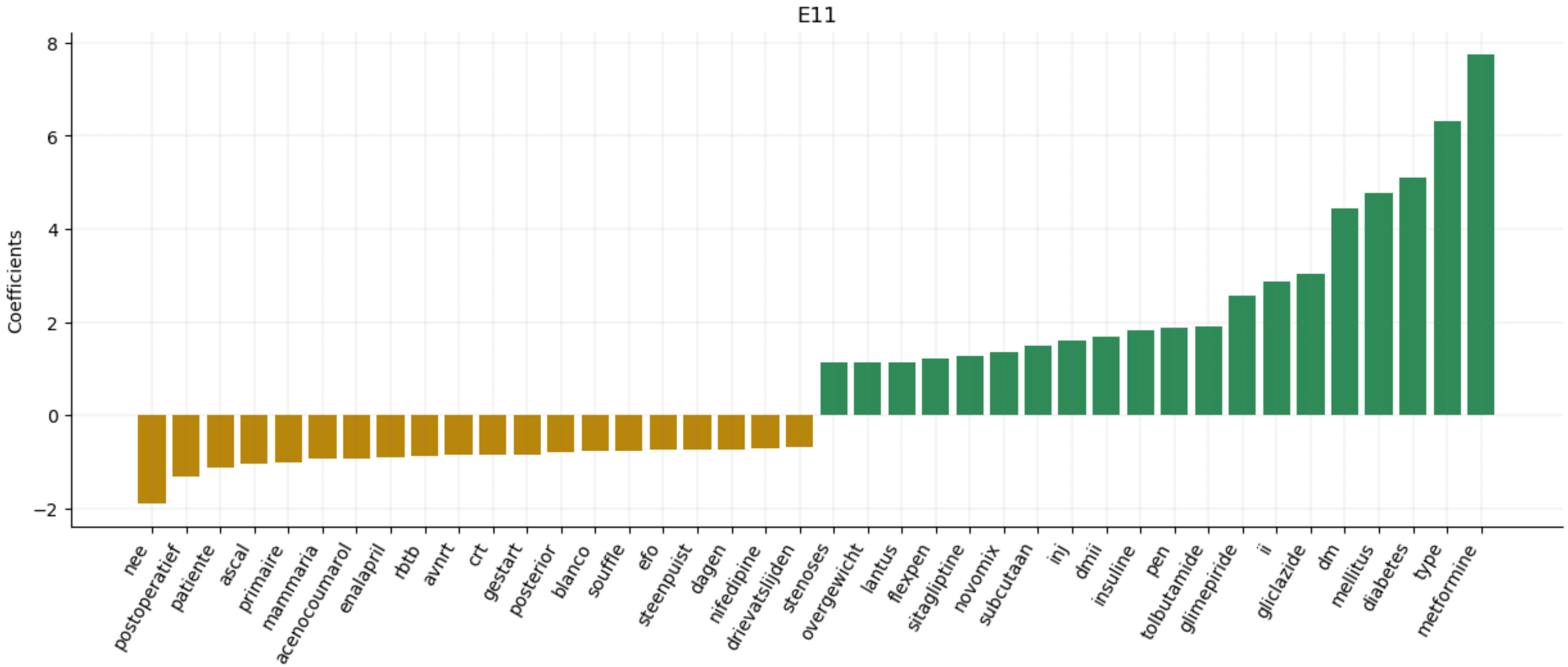
E11
Coefficients
8
6
4
2
0
−2
nee
postoperatief
patiente
ascal
primaire
mammaria
acenocoumarol
enalapril
rbtb
avnrt
crt
gestart
posterior
blanco
souffle
efo
steenpuist
dagen
nifedipine
drievatslijden
stenoses
overgewicht
lantus
flexpen
sitagliptine
novomix
subcutaan
inj
dmii
insuline
pen
tolbutamide
glimepiride
ii
gliclazide
dm
mellitus
diabetes
type
metformine

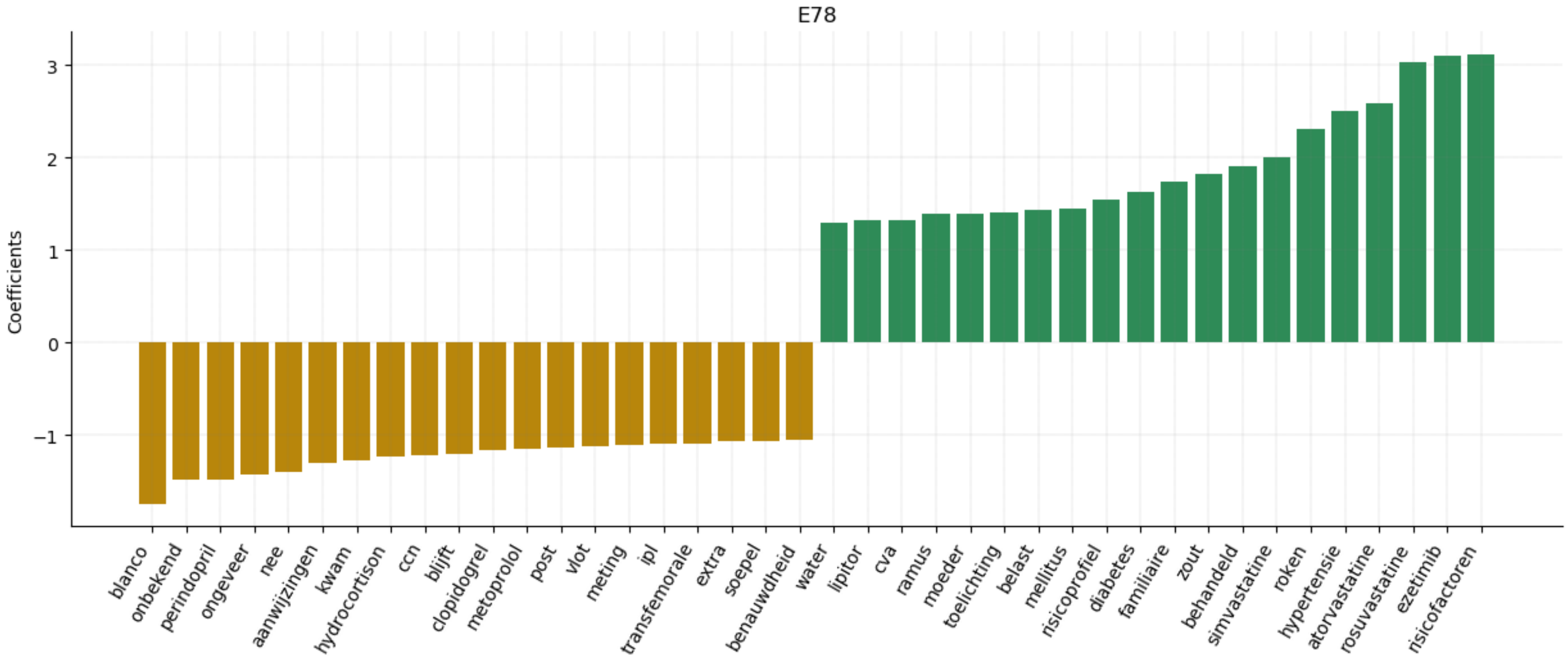
E78
Coefficients
3
2
1
0
−1
blanco
onbekend
perindopril
ongeveer
nee
aanwijzingen
kwam
hydrocortison
ccn
blijft
clopidogrel
metoprolol
post
vlot
meting
ipl
transfemorale
extra
soepel
benauwdheid
water
lipitor
cva
ramus
moeder
toelichting
belast
mellitus
risicoprofiel
diabetes
familiaire
zout
behandeld
simvastatine
roken
hypertensie
atorvastatine
rosuvastatine
ezetimib
risicofactoren

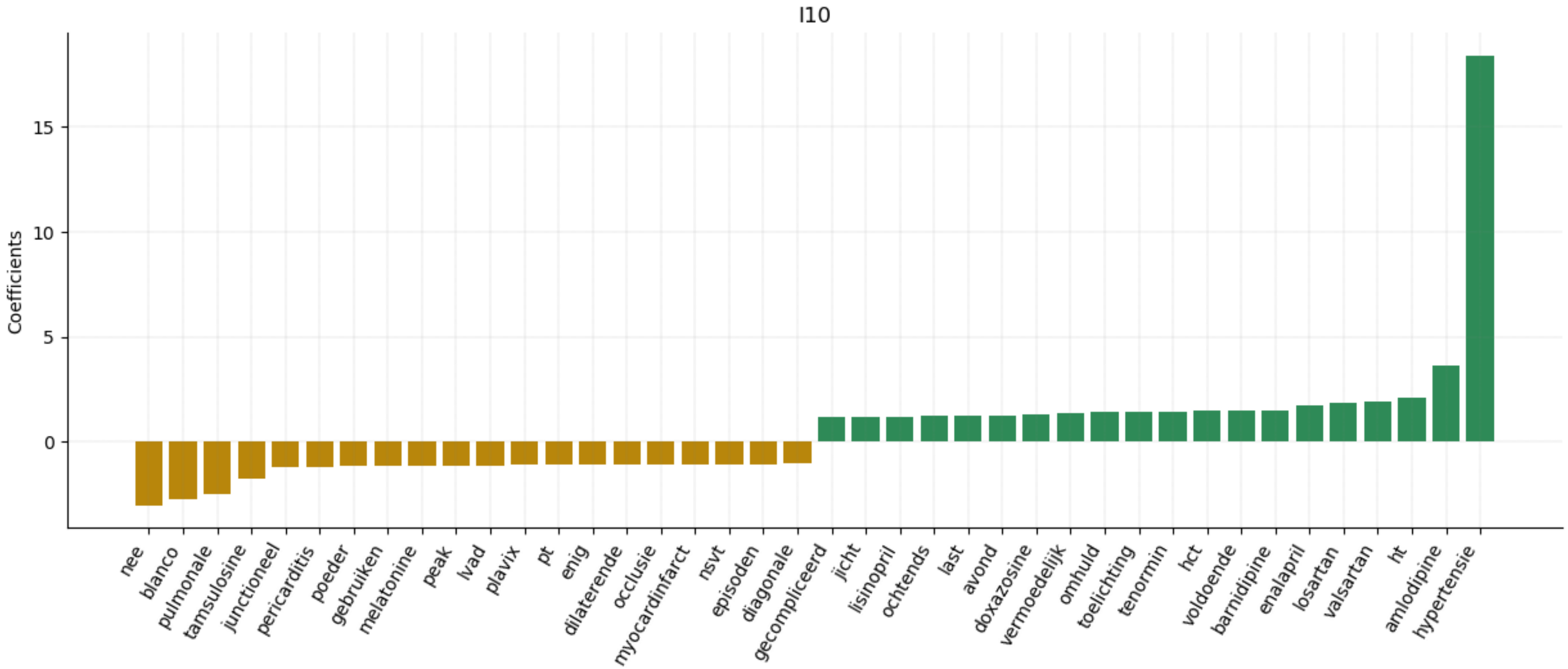
I10
Coefficients
15
10
5
0
nee
blanco
pulmonale
tamsulosine
junctioneel
pericarditis
poeder
gebruiken
melatonine
peak
lvad
plavix
pt
enig
dilaterende
occlusie
myocardinfarct
nsvt
episoden
diagonale
gecompliceerd
jicht
lisinopril
ochtends
last
avond
doxazosine
vermoedelijk
omhuld
toelichting
tenormin
hct
voldoende
barnidipine
enalapril
losartan
valsartan
ht
amlodipine
hypertensie

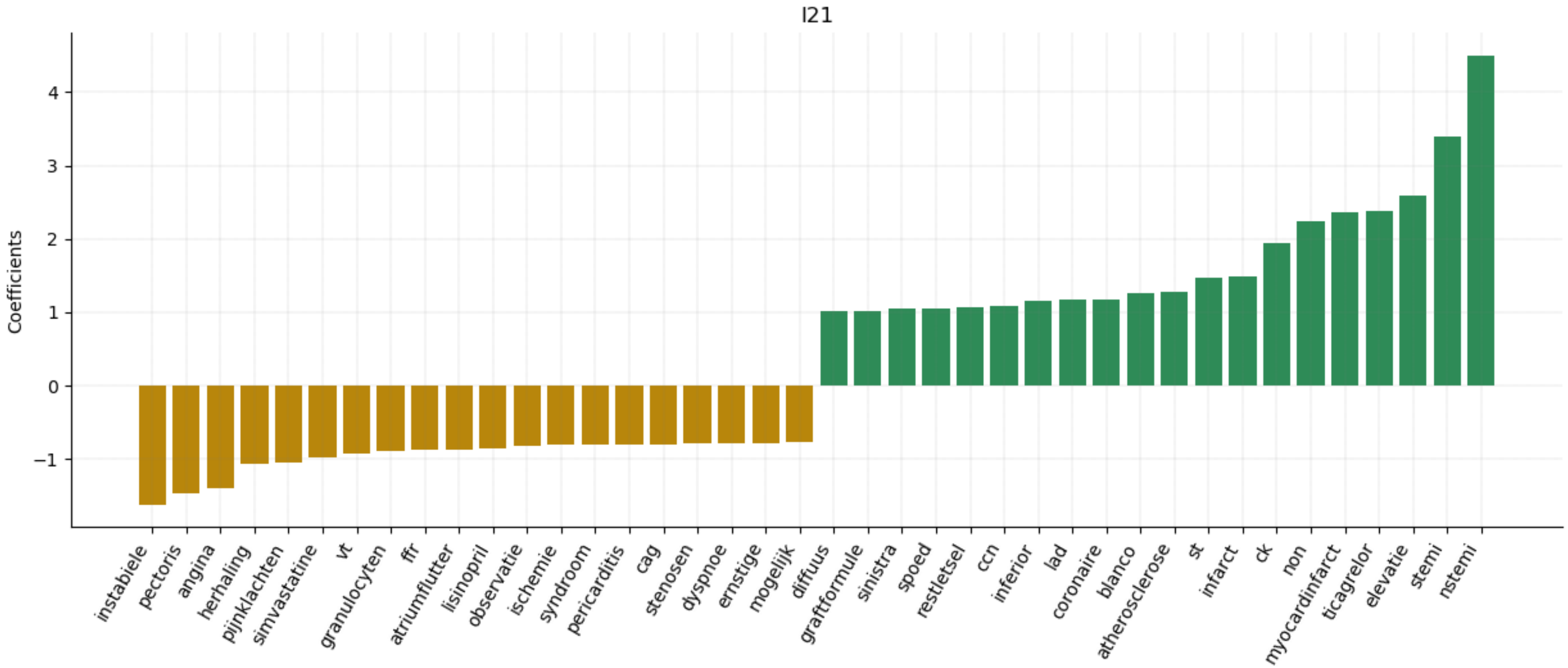
I21
Coefficients
4
3
2
1
0
−1
instabiele
pectoris
angina
herhaling
pijnklachten
simvastatine
vt
granulocyten
ffr
atriumflutter
lisinopril
observatie
ischemie
syndroom
pericarditis
cag
stenosen
dyspnoe
ernstige
mogelijk
diffuus
graftformule
sinistra
spoed
restletsel
ccn
inferior
lad
coronaire
blanco
atherosclerose
st
infarct
ck
non
myocardinfarct
ticagrelor
elevatie
stemi
nstemi

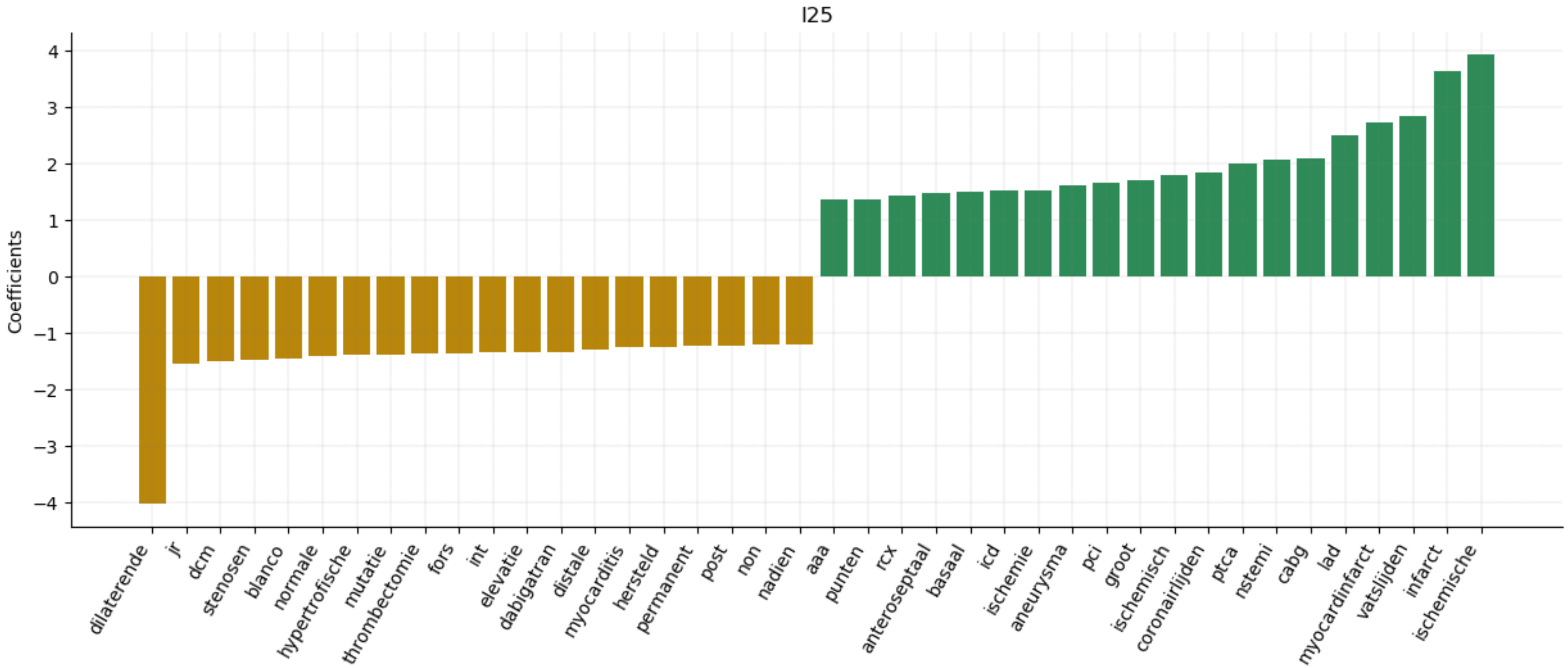
I25
Coefficients
4
3
2
1
0
−1
−2
−3
−4
dilaterende
jr
dcm
stenosen
blanco
normale
hypertrofische
mutatie
thrombectomie
fors
int
elevatie
dabigatran
distale
myocarditis
hersteld
permanent
post
non
nadien
aaa
punten
rcx
anteroseptaal
basaal
icd
ischemie
aneurysma
pci
groot
ischemisch
coronairlijden
ptca
nstemi
cabg
lad
myocardinfarct
vatslijden
infarct
ischemische

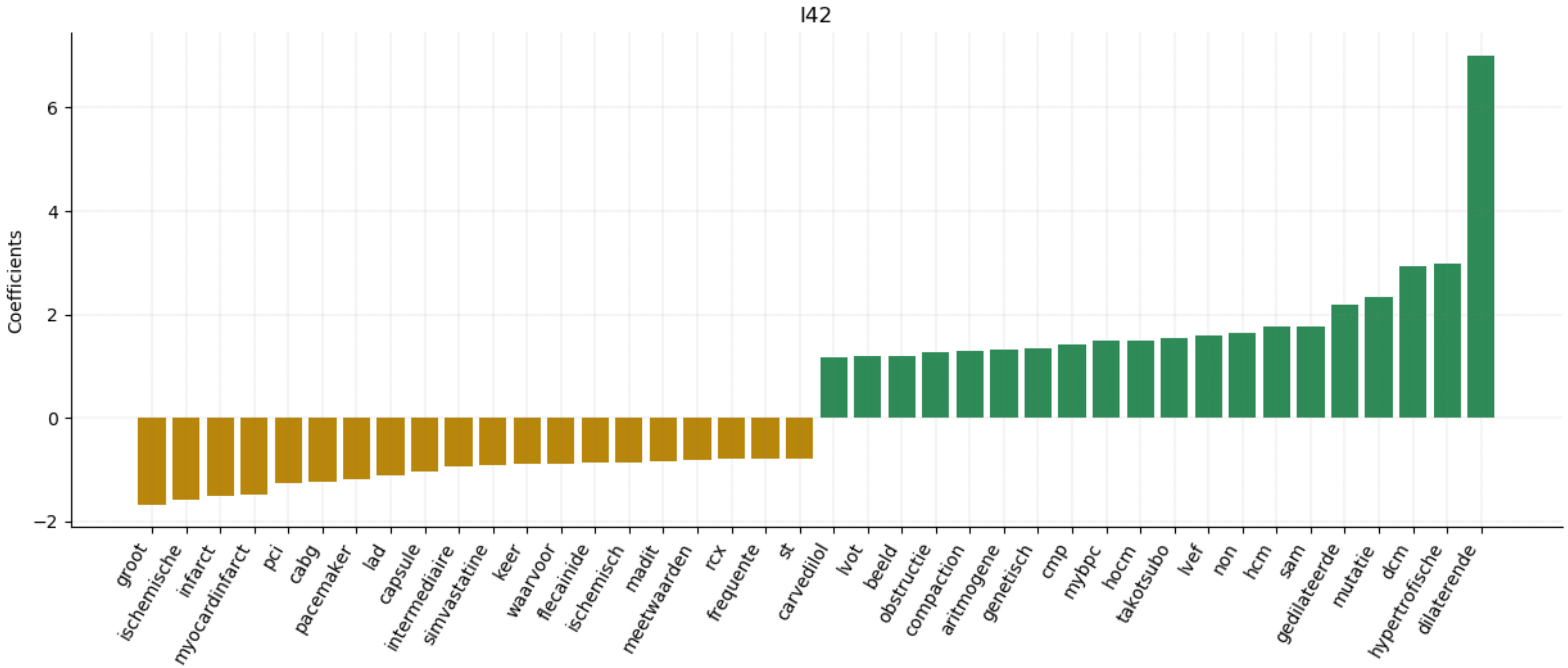
I42
Coefficients
6
4
2
0
−2
groot
ischemische
infarct
myocardinfarct
pci
cabg
pacemaker
lad
capsule
intermediaire
simvastatine
keer
waarvoor
flecainide
ischemisch
madit
meetwaarden
rcx
frequente
st
carvedilol
lvot
beeld
obstructie
compaction
aritmogene
genetisch
cmp
mybpc
hocm
takotsubo
lvef
non
hcm
sam
gedilateerde
mutatie
dcm
hypertrofische
dilaterende

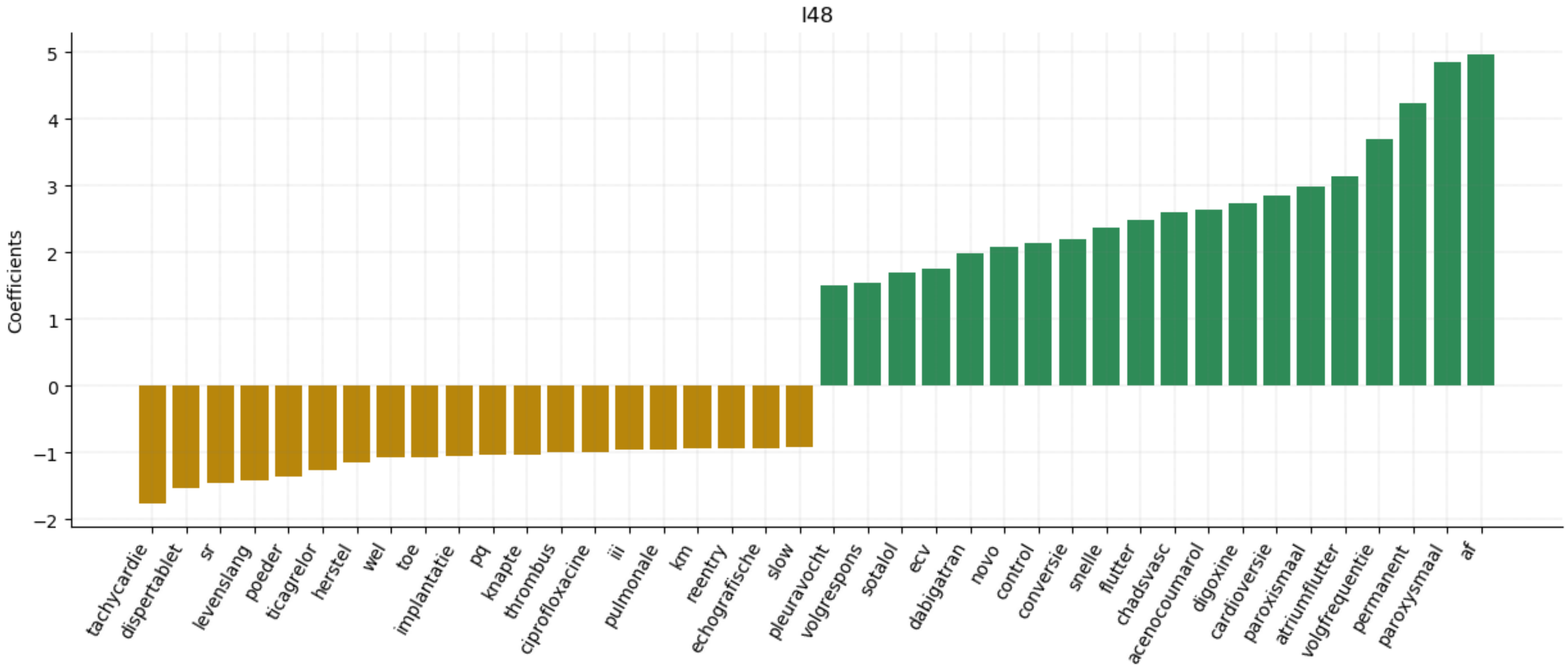
I48
Coefficients
5
4
3
2
1
0
−1
−2
tachycardie
dispertablet
sr
levenslang
poeder
ticagrelor
herstel
wel
toe
implantatie
pq
knapte
thrombus
ciprofloxacine
iii
pulmonale
km
reentry
echografische
slow
pleuravocht
volgrespons
sotalol
ecv
dabigatran
novo
control
conversie
snelle
flutter
chadsvasc
acenocoumarol
digoxine
cardioversie
paroxismaal
atriumflutter
volgfrequentie
permanent
paroxysmaal
af

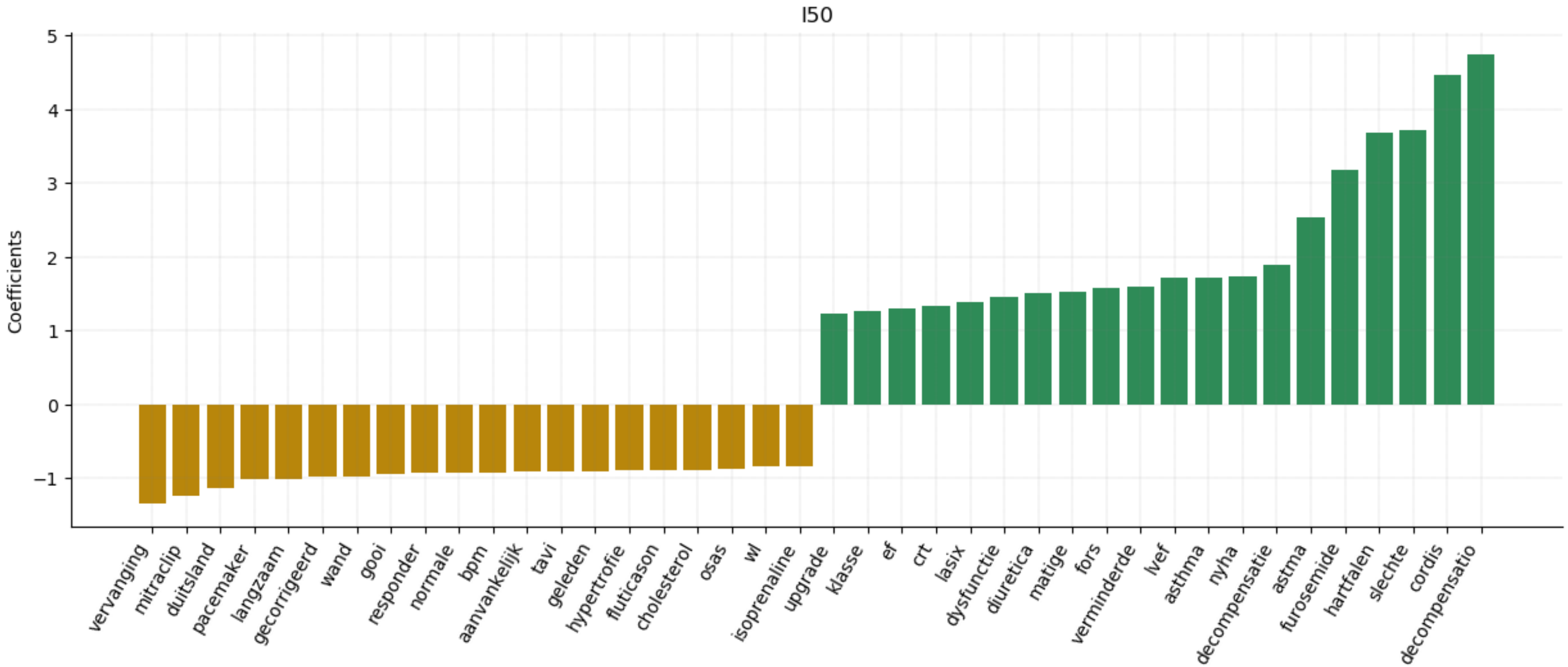
I50
Coefficients
5
4
3
2
1
0
−1
vervanging
mitraclip
duitsland
pacemaker
langzaam
gecorrigeerd
wand
gooi
responder
normale
bpm
aanvankelijk
tavi
geleden
hypertrofie
fluticason
cholesterol
osas
wl
isoprenaline
upgrade
klasse
ef
crt
lasix
dysfunctie
diuretica
matige
fors
verminderde
lvef
asthma
nyha
decompensatie
astma
furosemide
hartfalen
slechte
cordis
decompensatio

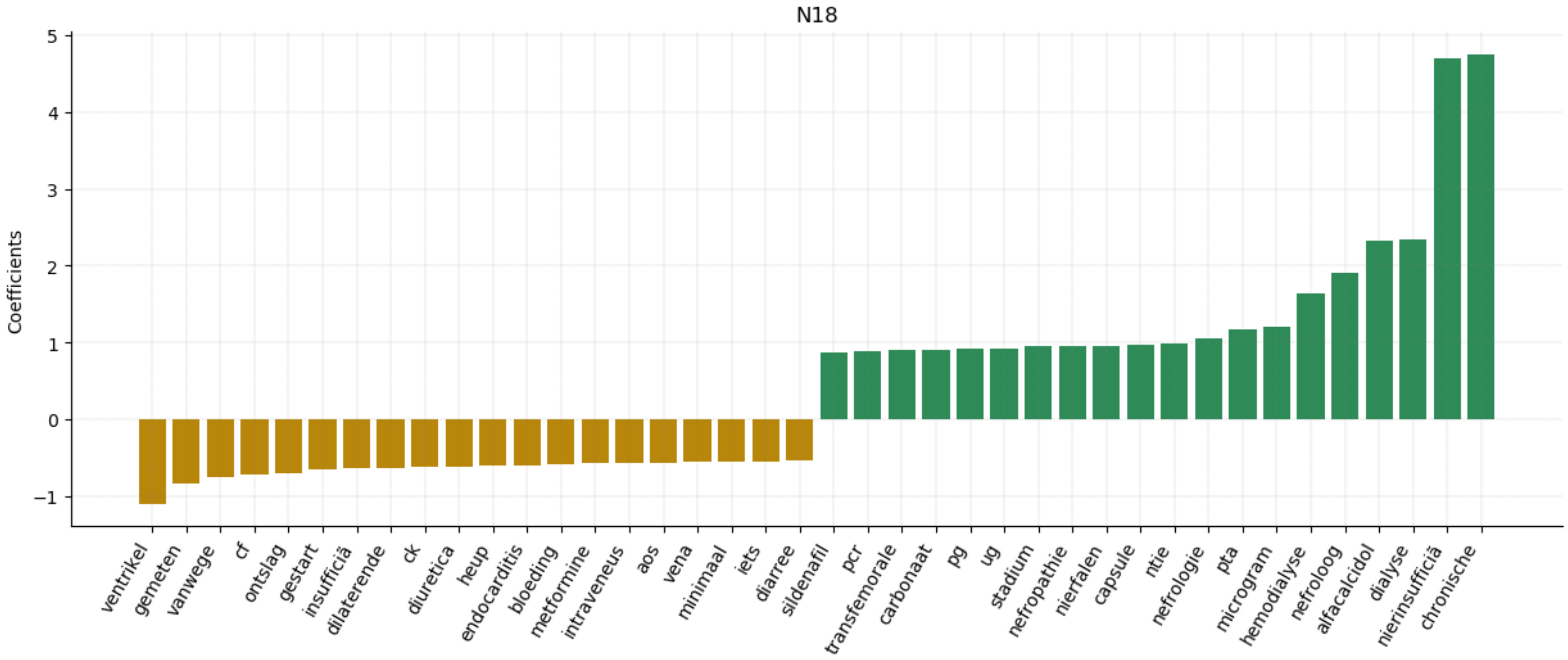
N18
Coefficients
5
4
3
2
1
0
−1
ventrikel
gemeten
vanwege
cf
ontslag
gestart
insufficiã
dilaterende
ck
diuretica
heup
endocarditis
bloeding
metformine
intraveneus
aos
vena
minimaal
iets
diarree
sildenafil
pcr
transfemorale
carbonaat
pg
ug
stadium
nefropathie
nierfalen
capsule
ntie
nefrologie
pta
microgram
hemodialyse
nefroloog
alfacalcidol
dialyse
nierinsufficiã
chronische
