# Supplementary material for: Automatic multilabel detection of ICD10 codes in Dutch cardiology discharge letters using neural networks
Source: NPJ Digit Med. 2021 Feb 26;4:37. doi: 10.1038/s41746-021-00404-9 (PMC7910461; doi:10.1038/s41746-021-00404-9)
Supplement: Supplementary file 2 — Reporting Summary [file 41746_2021_404_MOESM2_ESM.pdf]

nature research

Corresponding author(s): Arjan Sammani

Last updated by author(s): Dec 8, 2020

Reporting Summary

Nature Research wishes to improve the reproducibility of the work that we publish. This form provides structure for consistency and transparency in reporting. For further information on Nature Research policies, see our Editorial Policies and the Editorial Policy Checklist.

Statistics

For all statistical analyses, confirm that the following items are present in the figure legend, table legend, main text, or Methods section.

n/a	Confirmed	
☐	☒	The exact sample size (*n*) for each experimental group/condition, given as a discrete number and unit of measurement
☐	☒	A statement on whether measurements were taken from distinct samples or whether the same sample was measured repeatedly
☐	☒	The statistical test(s) used AND whether they are one- or two-sided<br>*Only common tests should be described solely by name; describe more complex techniques in the Methods section.*
☒	☐	A description of all covariates tested
☒	☐	A description of any assumptions or corrections, such as tests of normality and adjustment for multiple comparisons
☐	☒	A full description of the statistical parameters including central tendency (e.g. means) or other basic estimates (e.g. regression coefficient) AND variation (e.g. standard deviation) or associated estimates of uncertainty (e.g. confidence intervals)
☒	☐	For null hypothesis testing, the test statistic (e.g. *F*, *t*, *r*) with confidence intervals, effect sizes, degrees of freedom and *P* value noted<br>*Give P values as exact values whenever suitable.*
☒	☐	For Bayesian analysis, information on the choice of priors and Markov chain Monte Carlo settings
☒	☐	For hierarchical and complex designs, identification of the appropriate level for tests and full reporting of outcomes
☒	☐	Estimates of effect sizes (e.g. Cohen's *d*, Pearson's *r*), indicating how they were calculated

Our web collection on statistics for biologists contains articles on many of the points above.

Software and code

Policy information about availability of computer code

Data collection	SAS Enterprise Guide 8.2;
Data analysis	PyCharm 2020, R version 4.0.1 (2020). Python library used for BGRU: Keras. R-libaries used: DEDUCE, tm and tidytext. Code available via GitHub: https://github.com/bagheria/cardio-icd-assignment

For manuscripts utilizing custom algorithms or software that are central to the research but not yet described in published literature, software must be made available to editors and reviewers. We strongly encourage code deposition in a community repository (e.g. GitHub). See the Nature Research guidelines for submitting code & software for further information.

Data

Policy information about availability of data

All manuscripts must include a data availability statement. This statement should provide the following information, where applicable:

- Accession codes, unique identifiers, or web links for publicly available datasets
- A list of figures that have associated raw data
- A description of any restrictions on data availability

The dataset is not available due to patient privacy restrictions. However, the model may be shared upon request via the corresponding author.

Field-specific reporting

Please select the one below that is the best fit for your research. If you are not sure, read the appropriate sections before making your selection.

☒ Life sciences ☐ Behavioural & social sciences ☐ Ecological, evolutionary & environmental sciences

For a reference copy of the document with all sections, see nature.com/documents/nr-reporting-summary-flat.pdf

Life sciences study design

All studies must disclose on these points even when the disclosure is negative.

Sample size	Sample size not calculated. All available data was used for training and overfitting was avoided by using a dropout layer and recurrent dropouts at 0.2 rate. Based on results from train/test/validation they do not suggest overfitting.
Data exclusions	Data were not excluded
Replication	A train dataset was constructed on a random subset of 80% of training data. The classifiers were tested in remaining 20% of the data. Then, a non-overlapping temporal validation set was constructed. Further replication of this model may be allowed by contacting the corresponding author. As data are privacy sensitive, both model and data may not be published.
Randomization	A random subset of training data was selected 80% for the binary classification tasks
Blinding	As this is a computer model being trained, data blinding was not necessary. The medical coder was blinded however blinded for the outcome, for the calculation of Cohen's Kappa.

Reporting for specific materials, systems and methods

We require information from authors about some types of materials, experimental systems and methods used in many studies. Here, indicate whether each material, system or method listed is relevant to your study. If you are not sure if a list item applies to your research, read the appropriate section before selecting a response.

Materials & experimental systems

n/a	Involved in the study	
☒	☐	Antibodies
☒	☐	Eukaryotic cell lines
☒	☐	Palaeontology and archaeology
☒	☐	Animals and other organisms
☐	☒	Human research participants
☒	☐	Clinical data
☒	☐	Dual use research of concern

Methods

n/a	Involved in the study	
☒	☐	ChIP-seq
☒	☐	Flow cytometry
☒	☐	MRI-based neuroimaging

Human research participants

Policy information about studies involving human research participants

Population characteristics	Medical discharge notes of Dutch adult patients from the department of Cardiology were included in this study. A total of 5,548 was involved in the training dataset with a median age of 68 years (IQR 58 - 77) and 36% were female.
Recruitment	Patients were not actively recruited, as informed consent was not required by our medical ethical board for this retrospective cohort study. Patients that registered an opt-out were excluded from the analysis. Authors do not believe this affected the results.
Ethics oversight	University Medical Center Utrecht Medical Ethical Committee and Data Privacy Board

Note that full information on the approval of the study protocol must also be provided in the manuscript.
